# Assessment of vaccine candidates for persons aged 50 and older: a review

**DOI:** 10.1186/1471-2318-13-32

**Published:** 2013-04-15

**Authors:** Renske Eilers, Paul FM Krabbe, Ted GA van Essen, Anita Suijkerbuijk, Alies van Lier, Hester E de Melker

**Affiliations:** 1 Department of Epidemiology, University of Groningen, Academic Medical Center Groningen, P.O. Box 30.001, Groningen 9700 RB, The Netherlands; 2 Centre for Infectious Disease Control, National Institute for Public Health and the Environment (RIVM), P.O. Box 1, Bilthoven 3720 BA, The Netherlands; 3 Julius Center for Health Sciences and Primary Care, University Medical Center Utrecht, P.O. Box 85500, Utrecht 3508 GA, The Netherlands; 4 Center for Prevention and Health Services Research, National Institute for Public Health and the Environment (RIVM), P.O. Box 1, Bilthoven 3720 BA, The Netherlands

**Keywords:** Older persons, Vaccination, Vaccines, Herpes zoster, Pneumococcal disease, Pertussis, Hepatitis A

## Abstract

**Background:**

The increasing life expectancy in most European countries has resulted in growth of the population 50 and older. This population is more susceptible to infectious diseases because of immunosenescence, co-morbidity and general frailty. Thus, to promote healthy aging, vaccination against vaccine-preventable-diseases could be one strategy. In addition to its possible individual benefits, vaccination may also yield social benefits, such as a lower overall cost of healthcare. Most European countries, however, offer only influenza vaccine although vaccines for pneumococcal disease, herpes zoster, pertussis, and hepatitis A are also available. Our aim is to review the knowledge of these vaccines for persons aged 50 and older and explore the arguments for expanding current vaccination programmes beyond just influenza.

**Methods:**

The evaluation model of Kimman et al. was used to assess herpes zoster, pneumococcal disease, pertussis and hepatitis A in terms of four domains: pathogen, vaccine, disease outcomes and cost-effectiveness. The sources were Dutch surveillance systems, seroprevalence studies and the international literature.

**Results:**

Herpes zoster, pneumococcal disease and pertussis are prevalent among persons aged 50 and older. Vaccines vary in effectiveness and have mild and self-limiting side effects. Vaccination against pneumococcal disease and pertussis causes adaptation of the responsible pathogen. For pertussis and hepatitis A, the vaccine is not registered specifically for the elderly population. Vaccination against herpes zoster and pertussis could improve quality of life, while vaccination against pneumococcal disease and hepatitis A prevents mortality. However, only vaccination against herpes zoster and pneumococcal disease appear to be cost-effective.

**Conclusions:**

Vaccination can improve the health of the elderly population. As our review shows, however, the data are too incomplete to accurately judge its potential impact. More research is needed to determine how vaccination can most effectively improve the health of the growing population 50 years and older.

## Background

The increasing life expectancy in most European countries has resulted in the growth of the population 50 and older. Although this population will continue to increase, it may not age healthily [1,2]. It is estimated that by 2060, persons aged 65 and older will account for 30.0% of the total population of the EU-27 (the 27 Member States of the European Union), compared to 17.0% in 2008 [3].

As a result of immunosenescence (the gradual deterioration of the immune system), co-morbidity and general frailty, this population is more susceptible than younger persons to infectious diseases [4], resulting in higher mortality and morbidity rates in older persons than in young adults [5]. Infections may lead to irreversible frailty and, thereby, more dependency on long-term healthcare [6]. Vaccinating persons aged 50 and older against vaccine-preventable diseases (VPDs) may be one strategy to promote healthy aging.

Apart from possible benefits to individuals in this age group, vaccination may yield social benefits, such as lower overall costs of European healthcare. Most European countries offer influenza vaccine to this population, and some countries also offer pneumococcal vaccine [7]. However, vaccination against other VPDs is not common, despite initiatives to update the programmes [8].

The aim of this review is to evaluate the vaccines available for persons aged 50 and older and to explore the arguments of programmatic vaccination for herpes zoster, pneumococcal disease, pertussis and hepatitis A. For each of these infectious diseases, we review the current knowledge of these vaccines as specifically applied to this population and describe each of four domains (pathogen, vaccine, disease and cost effectiveness), according to the model of Kimman et al. [9].

### Rationale for vaccine choice

Herpes zoster, pneumococcal disease, pertussis and hepatitis A were chosen for this review because a vaccine for each is registered in Europe. Furthermore, a report by the Dutch health council on the future of the national immunization programme focused on the possibility of persons aged 50 and older as a target group for vaccination against herpes zoster, pneumococcal disease and pertussis. Their report considered vaccination for hepatitis A to be universal [10].

Beyond the Netherlands and Europe, the consensus in the United States is that these four vaccines are important throughout life. Vaccination for herpes zoster is recommended from the age of 60 and for pneumococcal disease from 65 by the Advisory Committee on Immunization Practices (ACIP) of the Centers for Disease Control and Prevention. Furthermore, a combined vaccine for tetanus, diphtheria and pertussis is recommended every 10 years. For hepatitis A, two doses in a lifetime are recommended when any risk factor is present [11].

## Methods

### Evaluation model

**Figure 1:**
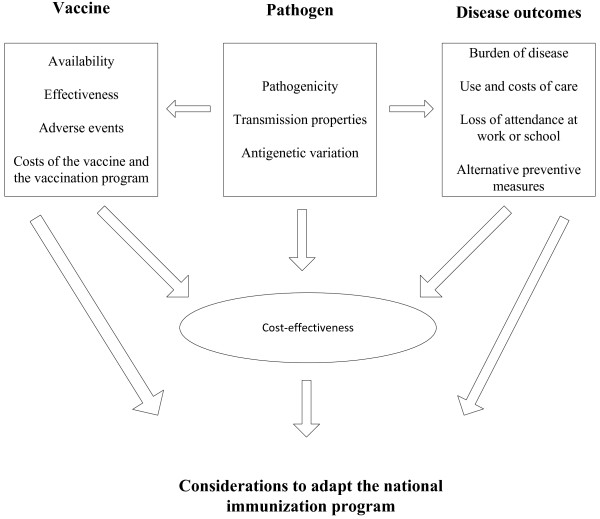
**Considerations in adapting the national immunization programme **[9]**.**

Vaccines against herpes zoster, pneumococcal disease, pertussis and hepatitis A were reviewed according to the criteria for adding new vaccines to the national immunization programme [9]. These criteria help in collecting relevant scientific information necessary for evaluation and decision making. The four domains of the model (pathogen, vaccine [as proposed by Kimman et al.], disease and cost-effectiveness) are presented as crucial elements of an adapted national immunization programme (Figure 1).

### Data source

To determine the incidence of herpes zoster during 1998–2009, information was assembled from the Continuous Morbidity Registration (CMR: 1998–2001) and the Netherlands Information Network of General Practices (LINH: 2002–2009), as well as the Dutch Institute for Health Services Research (NIVEL). The CMR and LINH are nationally representative networks of general practitioners (GP’s) that report the incidence of infectious diseases weekly.

We determined via notifications the incidence of pertussis since 1975 and hepatitis A since 1950 [12]. The incidence of invasive pneumococcal disease was based on isolates collected by The Netherlands Reference Laboratory for Bacterial Meningitis, a “laboratory-based surveillance system that collects nationwide pneumococcal isolates from blood and cerebrospinal fluid” and represents 25% of the Dutch population [13].

Information on hospital admissions for pertussis and herpes zoster was drawn from the National Medical Registration. Registration coverage of this system fluctuates around 90%. Mortality rates were obtained from Statistics Netherlands, which registers mortality data from death certificates on a statutory basis. Seroprevalence data to estimate prevalences of varicella zoster, pertussis and hepatitis A were available from two cross-sectional, population-based serosurveillance studies carried out in 1995–1996 and 2006–2007 among persons aged 0 to 79 years [14,15]. The international literature was searched for additional information on the four domains of the evaluation model for each of the infectious diseases in the study population.

## Results

### Pathogen

#### Pathogenicity

Nearly 100% of the Dutch population is seropositive for varicella zoster virus and thus at risk for the later development of herpes zoster [16]. The occurrence of herpes zoster and its recurrence is also elevated in persons with an immune deficiency [17].

For pneumococcal disease, the different serotypes vary in virulence, invasiveness and their potential to colonize. Of the more than 90 identified pneumococci serotypes, about 20% are responsible for more than 70% of the cases of invasive pneumococcal disease in all age groups [18]. By age, the incidence of invasive pneumococcal disease displays a parabolic pattern; the incidence is highest in children and persons aged 60 and older [19]. Nasopharyngeal carriage of *Streptococcus pneumoniae* was found to be low in non-institutionalized older persons (aged 65 and older) [20]. However, for certain serotypes, carriage was higher in older persons with much exposure to children compared to older persons with no exposure to children [20].

For pertussis, the incidence among persons aged 50 and older is rising. In 2006–2007 the reported incidence in persons aged 50 to 64 years old was 6 times higher than it was in 1995–1996, and the incidence was 8 times higher in those aged 65 to 79 years old in 2006–2007 compared to 1995–1996 [21]. Serological studies showed that between 6000 and 8000 per 100,000 persons aged 50 to 60 years and between 4000 and 6000 per 100,000 persons aged 60 to 79 years showed evidence of a recent (< 6 months) pertussis infection [22]. Pertussis is often atypical in adults. In the Netherlands, only 17% of persons 50 to 64 years old and 36% of those aged 65 to 79 years old who showed serological evidence of a recent pertussis infection had reported coughing in 2006–2007 [21].

For hepatitis A, it was estimated in 2012 that in the next 10 years 60% of the population 60 and older may be susceptible to that disease in the absence of vaccination or natural exposure [23]. Meanwhile, the average age of individuals who become infected is rising. Also in 2012, 12% of the participants in a seroprevalence study had been vaccinated against hepatitis A owing to travel. Because such a vaccination induces possible lifelong immunity, this group could be considered protected against hepatitis A even as they age [15]. The course of infection is more severe in persons aged 50 and older, and patients with chronic hepatitis B and C infections are at risk of more severe disease if they contract hepatitis A [24].

#### Infectiveness and transmissibility

Herpes zoster is the result of a reactivation of the dormant varicella-zoster virus in the central nervous system due to a diminished cell-mediated immunity. The vesicle rash specific to herpes zoster is contagious for non-immune persons until the vesicles have crusted; transmission occurs sporadically [25].

The transmission of pneumococci and pertussis is airborne, and both pathogens are contagious. Pneumococci are transmitted by nasopharyngeal carriers [26]. The infectiveness and transmissibility of pertussis is probably related to coughing, which makes pertussis highly contagious [27,28]. During an outbreak of pertussis in a Dutch religious institution, the attack ratio was 42% among the elderly [29].

Hepatitis A is transmitted by the fecal-oral route, by person-to-person contact or by ingestion of contaminated food or water [24]. Hepatitis A is also highly contagious due to the long survival period of the virus in the feces [24]. Community outbreaks in Latvia, Slovakia and in the Czech Republic showed a low attack rate among the elderly population [30-32]. To date, no outbreaks of hepatitis A have been reported among the elderly population in the Netherlands [33]. However, with the changing immunity profiles in the elderly population reflecting a decline in natural infection due to improved hygiene, these attack rates are expected to be higher in the future because of the diminishing of natural immunity.

#### Antigenic variation

Varicella zoster and hepatitis A are stable viruses. Whereas several genetic variations exist, all belong to the same known serotype [34,35]. However, many different serotypes of pneumococci are associated with invasive disease. A phenomenon called serotype replacement has been seen after routine pneumococcal vaccination: although herd-protection benefits for adults have been reported after the implementation of the 7-valent conjugate pneumococcal vaccine (PCV-7) for children, there has been a rise in pneumococcal disease in the study population. Its higher incidence is caused by non-vaccine PCV-7 serotypes. The serotype replacement depends on age, geographic region and the development of antibiotic resistance [18]. Similarly, for pertussis there is evidence that antigenic changes have occurred as a result of mass vaccination [36].

### Disease

#### Burden of disease

Herpes zoster, pneumococcal disease and pertussis are highly prevalent among persons aged 50 and older in the Netherlands [16,19,22,37]. Herpes zoster will develop in about 23% to 30% of the European population [38]. It causes a painful and itchy rash that proceeds to postherpetic neuralgia (PHN) in 9% to 34% of the elderly population who have the disease [39]. The pain associated with the rash and PHN is neuropathic and therefore less sensitive to painkillers, and there are no effective treatment options [40]. In the Netherlands, on average per year, 717 per 100,000 persons aged 50 and older consult a general practitioner because of herpes zoster, and 9.4 per 100,000 are admitted to the hospital. The number of hospital days increases with age. Herpes zoster has been reported to lead to a decrease in quality of life (health-related quality of life, HRQoL), with an impact on the activities of daily living (ADL) [41-44]. The total number of quality-adjusted life years (QALYs) lost because of herpes zoster has varied between 1060 and 3024 for persons aged 60 to 80 years [45].

Pneumococcal disease can lead to various clinical syndromes in older persons, such as invasive pneumonia (the most common), bacteraemia and meningitis [19]. The yearly average incidence of invasive pneumococcal disease is 54 per 100,000 persons aged 50 and older. For pneumonia, the incidence in general practice has been established at 17.5 per 1,000 patients aged 65 to 74 old and 31.4 per 1,000 patients aged 75 and older [46]. However, this overview included all causes of pneumonia, not just pneumococcal infection. It has been estimated that 30% to 40% of all pneumonia cases are caused by pneumococcal infection [47]. On average, 590 per 100,000 persons aged 50 and older are admitted to the hospital yearly for pneumococcal disease. The number of admissions to the intensive care unit are highest for meningitis, with 58% for persons aged both 50 to 64 years old and 65 and older, and lowest for bacteraemia, with 18% for both age groups. The average duration of a hospital admission was reported to be 13 days. Bacteraemia and meningitis were associated with the highest fatality rates, 40% and 39%, respectively, for persons aged 65 and older [19]. It is estimated that 648 QALYs are lost due to pneumococcal disease in the elderly population [48].

**Figure 2:**
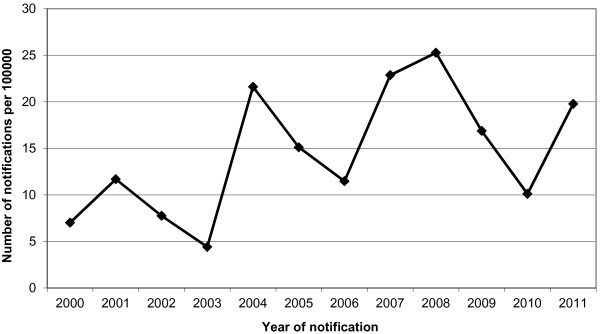
**Notifications for pertussis from 2000 to 2011 for persons aged 50 years and older.** The dots on the black line represent the number of notifications of pertussis per 100.000 persons 50 years and older for 2001 to 2012.

Pertussis causes symptoms similar to a common cold, but it can also lead to a prolonged chronic paroxysmal cough [39]. From 2004 on, a rise in the number of notifications has been observed for persons 50 aged and older (Figure 2). From 2002 to 2005, 4,963 cases were reported among persons aged 45 and older. In the same period, 10,338 GP consultations and 28 hospital admissions were reported among persons aged 45 and older [49]. Figure 2 shows an increase in the notifications of pertussis for persons aged 50 and older in 2000–2011. The complications of pertussis reported in adults are urinary incontinence, pneumonia, rib fractures, fainting, sinusitis and otitis media [50]. A pertussis outbreak in a monastery in the Netherlands showed the potential severity of the impact in the deaths of 4 persons aged 55 and older [29]. Pertussis also has an impact on the ADL [44]. We estimate that a symptomatic and an asymptomatic case represent a loss of 50 and 25 QALYs, respectively.^a^

Hepatitis A causes jaundice, nausea, fever and infection; very rarely it can lead to liver failure [24]. The severity increases with age, leading to a higher number of hospitalizations and to longer hospital stays [33]. The case fatality rate in persons aged 50 and older was reported to be 1.8% [51]. In total, 436 cases were reported among persons aged 50 and older from 2003 until May 2012. On average, the disease burden, as expressed in disability-adjusted life-years (DALYs), for all patients with notified cases of hepatitis A was estimated at 17 for 2005–2010 [33].

#### Use and cost of healthcare

Most of the cost of healthcare for the diseases under review is due to GP visits, hospital admissions, laboratory tests and medication such as antiviral medication for herpes zoster and antibiotics for pertussis and pneumococcal disease. For hepatitis A, there is no cure, but the symptoms can be controlled; additional expense is incurred to vaccinate the close contacts of those with hepatitis A.

For herpes zoster, it is estimated that the total cost in the Netherlands for GP consultations and drugs is €72.05 per case and €101.10 per case of PHN [45]. For pneumococcal disease, it is estimated that the mean direct cost per case (meaning the direct medical costs as registered by the Statistics Netherlands [CBS]), depending on the outcome of the infection, can vary from €17.10 (pneumonia requiring GP consultation) to €15,255.00 (meningitis). The mean indirect cost (meaning indirect costs of productivity losses registered by the CBS) per case varies from €6.50 (pneumonia requiring GP consultation) to €302.00 (meningitis) [48] (Table 1). For pertussis, it is estimated that the cost of healthcare was €75 per case for persons aged 45 and older in the Netherlands from 2002–2005. The largest contributing expenses were GP consultations and laboratory diagnostics [49]. For hepatitis A, no studies have been conducted on costs associated with the study population. The cost per patient, with an average age of 30 years, has been estimated at €1,579 [33].

**Table 1:** **Use and costs of healthcare for pneumococcal disease by disease outcome per case **[48]

	**Healthcare costs**	
**Disease outcome**	**Mean direct costs per case**^**a**^	**Mean indirect costs per case**^**a**^
Meningitis	€ 15,255.00	€ 302.00
Pneumonia requiring GP consultation	€ 17.10	€ 6.50
Pneumonia requiring hospitalization	€ 5,194.00	€ 173.00
Bacteraemia	€ 8,077.00	€ 197.00

^a^ The main direct and indirect costs were calculated by taking the age-related resource information published in Jansen et al. [19] and the direct medical costs and indirect costs of productivity losses registered by the Statistics Netherlands (//statline.cbs.nl, in Dutch).

#### Work absenteeism

The cost of absence from work is a growing concern in this age group. According to the CBS, 45% of the persons aged 55 to 65 years old were working in 2008. However, the proportion of persons who continue working has increased during recent years and will continue to grow [52].

Becoming infected while employed can lead to an employee’s loss of productivity. A study to estimate the productivity loss related to herpes zoster calculated that 64% of the persons aged 50 and older who had a job when they contracted the disease missed on average 43 hours of work; 76% reported on average 34 hours of decreased effectiveness [53]. For hepatitis A, the incidence is too low to account for lost productivity; no data on productivity loss was found for the other infectious diseases.

#### Alternative preventive measures

No alternative measures are known to prevent herpes zoster, pneumococcal disease or pertussis; only vaccination is effective. Practicing basic hygiene is important for preventing hepatitis A infection because of its fecal-oral transmission route, and post-exposure vaccination of patients’ close contacts has also proved effective in preventing hepatitis A [54].

### Vaccine

#### Availability of vaccines

The European organization for the registration of vaccines in Europe is the European Medicine Agency (EMA; http://www.ema.europa.eu/ema/).

Vaccines are available for herpes zoster, pneumococcal disease, pertussis and hepatitis A. Both Zostavax (for prevention of herpes zoster) and Prevenar13 (for prevention of pneumococcal disease) are registered in Europe for persons aged 50 and older. Pneumo23 is used in high-risk groups in the Netherlands [55]. Boostrix has been registered as a booster combination vaccine (DTaP-IPV) for the prevention of diphtheria (D), tetanus (T), pertussis (aP) and polio (IPV). Epaxal, Avarim and Havrix have been registered for the prevention of hepatitis A. The vaccines to prevent pertussis and hepatitis A have been licensed in Europe for the general adult population, not especially for the elderly population, although no upper age limit is given. In the United States, Boostrix was recently approved for use in persons aged 65 and older [56].

#### Effectiveness

**Figure 3:**
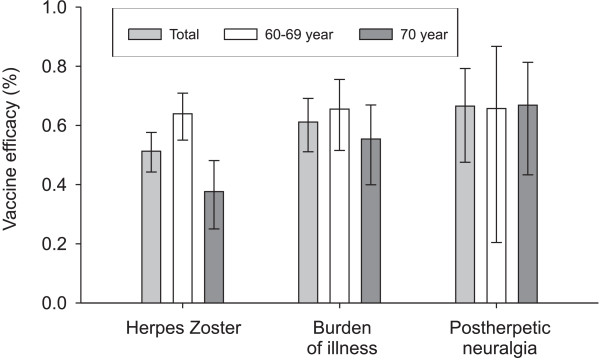
**The effectiveness of Zostavax for preventing HZ, burden of illness and PHN among different age groups **[38]**.** The light grey bar represents the vaccine effectiveness on the incidence of herpes zoster, the burden of illness and postherpetic neuralgia of the total study population. The white bar represents the vaccine effectiveness on the incidence of herpes zoster, the burden of illness and postherpetic neuralgia for those aged 60–69 years. The dark grey bar represents the vaccine effectiveness on the incidence of herpes zoster, the burden of illness and postherpetic neuralgia for those 70 years and older.

Zostavax has reduced the burden of herpes zoster by 61.1%, the incidence of PHN by 66.5% and the incidence of herpes zoster by 51.3%. These results were observed in persons aged 60 and older during five years of follow-up [57]. This vaccine’s effectiveness against herpes zoster was less in persons aged 70 and older than in those aged 60 to 69 years. The prevention of PHN was not age-dependent [57] (Figure 3). Vaccination has also been shown to reduce the negative impact of herpes zoster on the daily activities of persons aged 60 and older [58]. A single shot was found to confer protection for approximately six years, but more research on the duration of protection is needed [59].

Currently, a randomized controlled trial (RCT) is being performed to determine the effectiveness of Prevenar13 (a 13-valent conjugated vaccine) among persons aged 65 and older in the Netherlands [60]. This vaccine includes 65% to 77% of the serotypes responsible for invasive pneumococcal disease in Europe, whereas Pneumo23 covers 81% to 91% of the serotypes causing the disease in European adults [18]. Strong evidence of efficacy of a pneumococcal polysaccharide vaccine like Pneumo23 against invasive pneumococcal disease has been found, but the antibody levels in persons aged 65 and older seem to decline in three to five years [61,62].

Data are lacking on the effectiveness of pertussis vaccination in persons aged 50 and older. The risk of pertussis was reduced by 92% in patients aged 15 to 65 years old after acellular pertussis vaccination [63,64]. Since a single dose of Boostrix induced an effective immune humoral response against pertussis, it is likely to protect persons aged 55 and older. However, the immunogenicity in persons aged 75 and older was not clear because of the low number of subjects in the study [65]. Immunity in children after vaccination wanes within several years. This implies that one dose might lead to reduced transmission, but not to long-term individual protection [66]. Booster vaccinations may therefore be necessary.

One study was conducted on the immunogenicity of the hepatitis A vaccine in persons aged 50 and older. In this prospective uncontrolled study, an effective immune response was induced after two doses, achieving a protection of 98% [67]. Antibodies remain in vaccinated adults for 12 to 25 years, but their presence is potentially lifelong [68].

#### Adverse events, safety considerations

Adverse events following vaccination against herpes zoster, pneumococcal disease, pertussis and hepatitis A are commonly mild and self-limiting. More serious and long-term complications are rare (reported by http://www.fk.cvz.nl/).

Herpes zoster vaccine is contraindicated in persons with a hypersensitivity (allergy) to an active substance, immune deficiency, or untreated active tuberculosis or those who are being treated with immune suppressors or high doses of corticosteroids. The pneumococcal vaccine is contraindicated in people who are hypersensitive to the active substances, to any of the other ingredients or to diphtheria toxoid. People who have a high fever should not receive the vaccine until they have recovered. The pertussis vaccine is contraindicated in persons aged 50 and older who have an infectious disease at the time of vaccination. Hepatitis A vaccine is contraindicated in those who have a high fever as a consequence of disease, and Epaxal must not be given to persons aged 50 and older with a sensitivity to eggs and chicken protein.

Herpes zoster, 13-valent pneumococcal and pertussis vaccine have been given concomitantly with influenza vaccine and were well tolerated [69-71]. Boostrix has also been given concomitantly with Havrix and was well tolerated [72]. To achieve an optimal benefit, pneumococcal vaccination and herpes zoster vaccination must be given four weeks apart [73].

#### Costs of the vaccine and the vaccination programme

Market prices for the vaccines range from €14.98 (Pneumo 23) to €137.33 (Zostavax) [74]. The prices are likely to be lower when the products are used in a vaccination programme. In addition, the number and frequency of doses as well as the expense of administration will influence the costs of a vaccination programme.

The estimated cost of implementing a vaccination programme, including administration costs, in the Netherlands for herpes zoster within the current influenza programme ranges from roughly €14.7 million for persons aged 60 and older to €4.9 million for persons aged 80 and older (assuming an uptake of 75%). Furthermore, once-only costs would be €0.3 million [45].

For hepatitis A, the costs of a vaccination programme, including a catch-up programme for all persons aged 60 and older performed by general practitioners, would be €210 million in the first year and €13 million in subsequent years (indexed for 2009 for all persons aged 60 and older).

We estimated the annual costs of pertussis (with Boostrix) and pneumococcal vaccination (with Prevenar13), assuming an uptake of 75% among persons aged 60 and older and at the retail price.^b^ For pertussis vaccination, an indication of the annual costs would be €65 million; for pneumococcal vaccination, it would be €190 million. This estimate does not include additional costs, such as the fee for general practitioners or the potential need for revaccinations.

### Cost-effectiveness

A Dutch cost-effectiveness study found that herpes zoster vaccination for persons aged 70 years old was marginally cost-effective (€21,716 per QALY, based on the reduction in burden of disease). Vaccination of other age groups was not cost-effective owing to the vaccine’s cost and lack of effectiveness [45].

The cost-effectiveness of the 13-valent pneumococcal vaccine was demonstrated in different scenarios [48]. No cost-effectiveness study is available on pertussis vaccination of the study population. A world-wide review of economic evaluations of booster vaccination against pertussis for adults concluded that many studies were strongly affected by the lack of reliable input data on real incidence and by the number of unreported cases [75]. A study from Germany concluded that vaccinating adults with a booster vaccination (>18 years old) would be cost-effective when the incidence is greater than 120 per 100,000 population [76]. It was found unlikely that vaccination of the study population against hepatitis A in the Netherlands would be cost-effective due to the currently low incidence rates. Moreover, most cases in the Netherlands are related to travel, and an increasing number of persons are being vaccinated against hepatitis A [23].

A summary of the results discussed above is given in Table 2.

**Table 2:** **An overview of the model of Kimman et al. **[9]** for herpes zoster, pneumococcal disease, pertussis and hepatitis A**

	**Pathogen**		**Disease**				**Vaccine**						**Cost-effectiveness**
	**Bacteria or virus**	**Clinical picture**	**Age-related risk**	**Morbidity**		**Mortality**	**Efficacy**	**Side effects**	**No. of doses**	**Period of protection**	**Administration with other vaccines**	**Costs + reimbursement (in the Netherlands)**	
				**GP consultation (incidence)**	**Hospital admission (incidence)**								
Herpes zoster	Herpes zoster virus (reactivation of varicella zoster virus)	Painful itchy rash, PHN	Risk increases with age50% >85 years	717/ 100,000 (≥50 years)	9.4/100,000 (≥50 years)	Low	51.3% -66.5%	Mostly local symptoms and headaches	1	6 years?	Influenza vaccine. Zostavax and pneumococcal vaccine should be given 4 weeks apart	€43.91/137.33, no reimbursement	Marginal
Pneumococcal disease	Staphylococcus pneumoniae	Pneumonia, Meningitis, Bacteraemia	Incidence rises with age	-	590/100,000 (>50 year)	20%	PPV23: strong indication for preventing IPD Prevenar13: currently investigated	Mostly local symptoms and headaches	2?	3-5 years?	Influenza vaccine, Zostavax and pneumococcal vaccine should be given 4 weeks apart	Prevenar13: €67.72, no reimbursement Pneumo23: €14.97-, reimbursementunder certain circumstances	Yes
Pertussis	Bordetella pertussis	Influenza-like symptoms and chronic paroxysmal cough	Pertussis becomes more prevalent among those ≥50 years	10,388 >45 years 2002-2005	28 >45 years 2002-2005	Low	Secondary vaccine failure is present in older persons	Mild symptoms	Possible multiple	4-12 years in children	Havrix may be given simultaneously	€21.18, no reimbursement	Unknown
Hepatitis A	Hepatitis A virus	Jaundice, fever, nausea, vomiting, liver failure	Aging increases susceptibility	Morbidity increases with age		1.8%	Epaxal: 100% after 2 doses	Mostly local symptoms, headaches and fatigue	2	12-25 years, possibly lifelong	Boostrix may be given simultaneously	Epaxal and Havrix: €21.63-32.45, no reimbursement	Highly unlikely

## Discussion

We assessed herpes zoster, pneumococcal disease, pertussis and hepatitis A vaccines according to a previously formulated set of criteria for additions to a national immunization programme. Herpes zoster, pneumococcal disease and pertussis are common among persons aged 50 and older in the Netherlands, and in the future, this study population will become more susceptible to hepatitis A, because fewer persons will have acquired natural immunity through exposure. For each of these infectious diseases, a vaccine is available, although its effectiveness varies. Whereas vaccination might have consequences for the virulence and prevalence of pneumococcal disease and pertussis, it could lower the disease burden and reduce healthcare costs. Only vaccination against herpes zoster and pneumococcal disease currently appear to be cost-effective.

Routine vaccination in childhood has been extremely successful in reducing the health burden, as shown in numerous studies. Our review suggests that, although vaccination can improve the health of persons aged 50 and older, the data, even on available vaccines, is still far from sufficient to judge the full potential impact. Besides, the vaccines against pertussis and hepatitis A have not yet been registered for the study population in Europe.

Vaccination against certain types of pathogens will prevent mainly severe disease and death, such as in invasive pneumococcal disease, where mortality is the main concern. Vaccination against other types (with low case fatality rates) will increase a person’s quality of life by limiting functional loss, reducing frailty and preventing the exacerbation of the underlying disease, such as in herpes zoster and pertussis, where loss of quality of life is most prominent [9]. The effect of vaccination can also differ across age groups. For the herpes zoster vaccine, persons aged 60 to 69 years profit more from a decrease in the incidence of the disease and its burden than persons aged 70 to 79 years. However, the effectiveness of the vaccine in regard to the incidence of PHN hardly differs between persons aged 60 to 69 years and those aged 70 to 79 years.

Considering the incidence and the crude estimated burden of the various diseases, prevention of pneumococcal disease and herpes zoster might warrant higher priority than prevention of pertussis and hepatitis A. The study population will become more susceptible to hepatitis, yet they might avoid it by being vaccinated for travel. This protection is possibly life long, thereby making vaccination of the study population against hepatitis less of a priority. In the seroprevalence report of 2005–2006, already 23% of the persons aged 0 to 79 years old had been vaccinated against hepatitis A. For pertussis, however, there may be much underreporting, which is suggested by the difference between notifications and serological incidence, so the burden of disease is unclear. Lack of information on the disease burden makes it hard to compare pertussis to influenza, which is already in the vaccination programme for the study population in some European countries [7].

Vaccination of persons aged 50 or older is important, considering immunosenescence and the fact that infections frequently lead to irreversible frailty and thereby to more dependency after recovery [6]. Therefore, insight into the optimal timing of vaccination is essential. Vaccination at the age of 50, before immunosenescence occurs, might provide increased protection [77]. For that reason, we took 50 years old as the minimum age for this review. Of note, the lowest age at which most European countries offer influenza vaccination is 60. Thus, a possible reduction of the age for access to the influenza programme might be beneficial; that possibility is being considered by the health council of the Netherlands [78].

Furthermore, decisions on vaccination in childhood should take into account the potential change in disease dynamics in the study population and vice versa. For example, routine vaccinations against varicella in children could have implications for the occurrence of herpes zoster in persons aged 50 and older [79].

Other questions remain about the effectiveness and duration of protection of the candidate vaccines in the study population. In one trial involving herpes zoster vaccine, its efficacy was the lowest for persons aged 70 and older; this was the group with the highest incidence and disease burden [57]. For pneumococcal disease, clarification of the effectiveness of the 13-valent conjugated vaccine in persons aged 65 and older are pending the results of an RCT [60]. Furthermore, questions remain about the number of doses needed to obtain an optimal and lasting immune response and the necessity of repeating the vaccination.

One of the major questions concerning potential improvements through vaccination in the health of persons aged 50 and older is whether they would accept such an intervention. This population may perceive the situation differently from younger individuals. For example, in the United States, vaccination against herpes zoster is recommended, but the uptake is low in persons aged 60 and older [80]. Before the implementation of new vaccination strategies for the study population, it is of utmost importance to gain more knowledge about acceptance of new vaccines, its uptake and the determinants that influence the uptake. Research should also address the preferences of the study group with respect to the effects and implications of primary prevention for VPDs.

## Conclusion

In conclusion, in the field of primary prevention for persons aged 50 and older, vaccination against herpes zoster and pneumococcal disease are potentially effective. It should be kept in mind that, although we have mainly used Dutch data, certain aspects of vaccination are not limited to a specific country. Therefore, we believe that the results of this review are also relevant to other Western European countries. More research would ensure the best possible improvement in health for the growing population of persons aged 50 and older.

## Consent

Written informed consent was obtained from the patient for publication of this report and any accompanying images.

## Endnotes

^a^Based on cases reported in CMR during the period 2002–2005 and the weight estimates given by Lee et al., 2007 (72).

^b^Retail price Boostrix: €23.29.

Retail Prevenar13: €67.72.

Assuming uptake of 75%: 3,700,000 × 0.75 = 2,800,000 persons aged 60 and older (Central Office for Statistics Netherlands).

## Abbreviations

EU-27: The 27 Member States of the European Union; VDP: Vaccine-preventable diseases; GP’s: General practitioners; CMR: Continuous Morbidity Registration; LINH: Netherlands Information Network of General Practices; NIVEL: Dutch Institute for Health Services Research; PCV-7: 7 valent conjugated pneumococcal vaccination; DTaP-IPV: Diphtheria, tetanus, pertussis and polio; RCT: Randomized controlled trial; PHN: Postherpetic neuralgia; ADL: Activities of daily living; QUALY: Quality-adjusted life years; DALY: Disability-adjusted life-years; CBS: Statistics Netherlands.

## Competing interest

G.A. van Essen received travel costs for a speech for Abbott.

## Authors’ contributions

RE gathered the literature, drafted and wrote the manuscript, PK and GE critically reviewed the manuscript, AS en AvL provided data for the manuscript and critically reviewed the manuscript, HM contributed to the design of the manuscript and critically reviewed the manuscript. All authors read and approved the final manuscript.

## Pre-publication history

The pre-publication history for this paper can be accessed here:

http://www.biomedcentral.com/1471-2318/13/32/prepub
